# Dietary Regulation of Hepatic Triacylglycerol Content—the Role of Eucaloric Carbohydrate Restriction with Fat or Protein Replacement

**DOI:** 10.1016/j.advnut.2023.08.005

**Published:** 2023-08-15

**Authors:** Anne-Marie Lundsgaard, Kirstine Nyvold Bojsen-Møller, Bente Kiens

**Affiliations:** 1 Section of Molecular Physiology, Department of Nutrition, Exercise and Sports, University of Copenhagen, Copenhagen, Denmark; 2 Department of Endocrinology, Copenhagen University Hospital Hvidovre, Hvidovre, Denmark

**Keywords:** NAFLD, MAFLD, fatty liver, diet, steatosis, carbohydrate, fatty acids, fatty acid oxidation

## Abstract

Accumulation of hepatic triacylglycerol (TG) is highly associated with impaired whole-body insulin-glucose homeostasis and dyslipidemia. The summarized findings from human intervention studies investigating the effect of reduced dietary carbohydrate and increased fat intake (and in studies also increased protein) while maintaining energy intake at eucaloric requirements reveal a beneficial effect of carbohydrate reduction on hepatic TG content in obese individuals with steatosis and indices of insulin resistance. Evidence suggests that the reduction of hepatic TG content after reduced intake of carbohydrates and increased fat/protein intake in humans, results from regulation of fatty acid (FA) metabolism within the liver, with an increase in hepatic FA oxidation and ketogenesis, together with a concomitant downregulation of FA synthesis from de novo lipogenesis. The adaptations in hepatic metabolism may result from reduced intrahepatic monosaccharide and insulin availability, reduced glycolysis and increased FA availability when carbohydrate intake is reduced.

**Statement of Significance**

Dietary carbohydrate reduction and increased fat intake under eucaloric weight maintenance conditions can lead to a clinically relevant reduction in liver fat, associated with the regulation of fat metabolism within the liver, at the level of ketogenesis and de novo lipogenesis.

## The Importance of the Liver in the Metabolic Syndrome

Nonalcoholic fatty liver disease (NAFLD) is defined by fat accumulation in the liver (steatosis) exceeding 55 mg/g liver (5.5%) and in the absence of excessive alcohol intake, although the definition of excess liver fat content may vary dependent on assessment method [1]. Excess accumulation of fat in the liver is associated with dysregulated whole-body glucose and insulin homeostasis [2], as well as dyslipidemia [3], and the recognition of the association to metabolic comorbidities has led to the proposal of a new nomenclature: metabolic dysfunction associated fatty liver disease [4]. A relationship is thus described between the degree of hepatic triacylglycerol (TG) accumulation and the occurrence and severity of metabolic syndrome components [[5], [6], [7]], elevated hepatic glucose production [7,8], and reduced insulin clearance by the liver [9]. Notably, in a cross-sectional study of 352 individuals, elevated glucose production and increased plasma TG were observed already at hepatic TG levels exceeding 2% [10].

Approximately 30% of the global population is estimated to have NAFLD [11]. However, the actual prevalence may be even higher with increased diagnostic testing, as there is a lack of reliable and applicable diagnostic tests [12]. Notably, the highest prevalence of NAFLD is found in individuals with diabetes [13].

Higher BMI (overweight or obesity) is also a dose-dependent risk factor for fatty liver [14], whereas it should be recognized that up to 20% of individuals with steatosis have normal weight [11]. The etiology of excess liver TG is likely multi-factorial, and is believed to result from a combination of increased extrahepatic plasma fatty acid (FA) availability, altered intrahepatic FA oxidation and VLDL-TG kinetics, together with increased intrahepatic de novo lipogenesis (DNL) [[15], [16], [17]]. The relative importance of the respective mechanisms likely depends on the metabolic context.

Reduction of excess hepatic TG content is an appealing therapeutic goal applying to a significant proportion of the population that targets both potential metabolic dysregulation as well as risk of liver disease. There is no specific pharmacological treatment for NAFLD, and weight reduction is the cornerstone of treatment [18]. A significant weight loss may, however, for many individuals with obesity be difficult to achieve and maintain [19] and may not be the obvious choice for the nonobese population with NAFLD. Thus, it is of clinical relevance to investigate the effect of eucaloric dietary regimens on the regulation of liver TG content. The nutritional approach to NAFLD has been debated for decades, and there is still a lack of consensus regarding whether carbohydrate restriction or low-fat regimens should be applied in the treatment or prevention of NAFLD, and also the role of protein intake is not clear [[20], [21], [22], [23], [24], [25], [26]]. The current international recommendations for carbohydrate intake are in the range of 45–65E% [27], and this leaves absolute carbohydrate intake in the range of 225–470 g assuming a daily energy intake of 2000–3000 kcal.

Herein, the effect of reduction of dietary carbohydrate intake and replacement by fat or protein on hepatic TG content assessed by ^1^H-magnetic resonance spectroscopy (MRS) will be summarized and discussed, with emphasis on human intervention studies which per design aimed to meet eucaloric energy requirements. The presence (or lack) of steatosis, obesity, and insulin resistance will be considered as all could modify the response to changes in carbohydrate intake. An important scope of this review is to provide an in-depth discussion, from evidence in humans, of adaptations in hepatic substrate availability and metabolism when carbohydrate intake is reduced, and fat (or protein) is increased. The focus will be on total macronutrient content, as the role of carbohydrate and fat type will be beyond the limitations of this review, and has been covered by other recent reviews [28].

## Reduced Total Calorie Intake compared with Carbohydrate or Fat Reduction

Under hypocaloric conditions, it has been shown that dietary carbohydrate reduction was superior to fat reduction when it comes to lowering of hepatic TG content. In obese men and women with steatosis and insulin resistance measured by elevated HOMA-IR index, a 2-d 1000-kcal restricted diet with 10 energy% (E%) carbohydrate (≤50 g/d) and 75E% fat led to a 30% decrease in hepatic TG content (11–8%) in one group compared with a 9% decrease in liver TG (12–11%) after intake of 65E% carbohydrate (∼180 g/d) and 20E% fat in the other group, with a similar average weight loss of 1.6 and 2.2 kg, respectively [29]. These findings suggest that reduced carbohydrate intake may be more important for the regulation of liver TG content than energy restriction. One study aimed to assess the role of carbohydrate restriction compared with calorie restriction. In a parallel intervention, the effect of 2 wk reduced carbohydrate intake with ad libitum calorie intake compared with reduced total calorie intake were compared in obese men and women with steatosis and elevated fasting plasma glucose [30]. Self-reported carbohydrate intakes were 26 ± 8 g/d (8E% carbohydrate and 59E% fat) and 169 ± 33 g/d (50E% carbohydrate and 34E% fat) in the carbohydrate- and calorie-restricted groups, respectively, and hepatic TG content decreased more with reduced carbohydrate (22 ± 13–10 ± 7%) compared with reduced calorie intake (19 ± 10–14 ± 7%) [30]. Limiting conclusions from the study, a similar weight loss of ∼4 kg was observed in both the groups, indicating a negative energy balance also in the carbohydrate-restricted group. However, the studies by Kirk et al. [29] and Browning et al. [30] may suggest that reduced carbohydrate (and increased fat intake) may be more important in the regulation of hepatic TG content than energy restriction.

Observations in cross-sectional studies with registration of the habitual diet support this notion. In these studies, positive associations were obtained between habitual carbohydrate intake, but not energy intake, and the degree of steatosis assessed from liver biopsies in 74 severely obese men and women [31] and in 82 obese adolescents with liver TG assessed from ultrasonography [32]. Also in 24 overweight men and women with steatosis, a positive correlation between habitual carbohydrate intake and biopsy-evaluated hepatic TG content was obtained [33]. The range of habitual carbohydrate intake was from 30E% to 60E%.

To understand the impact of dietary carbohydrate replacement by fat (or protein) on hepatic TG content, well-controlled intervention studies in which energy intake is maintained at eucaloric requirements are essential. In the following, such intervention studies in individuals with elevated liver fat are summarized.

## Regulation of Hepatic TG Content by Reduced Carbohydrate Intake at Eucaloric Conditions

Eucaloric intervention studies with carbohydrate replacement have been conducted primarily in middle-aged overweight and obese individuals with steatosis (Table 1). In 5 of the interventions, carbohydrates were replaced by fat [[34], [35], [36], [37], [38]], whereas 6 studies replaced carbohydrates by a combination of increased fat and increased protein content (increased protein of 22–30 E%) [[39], [40], [41], [42], [43], [44]].

**TABLE 1:** Eucaloric dietary intervention studies applying H-MRS and manipulating carbohydrate intake while using fat or protein replacement

Study	Design	Baseline liver TG	Diets	Weeks	Body weight	Liver TG change	Plasma parameters
[37]	Crossover *n* = 10 (F) 43 ± 5 y (SD) BMI 33 ± 4 Average HOMA-IR 2.6	10 ± 7%	LC CHO 31E%, FAT: 56E%, PRO 13E% SFA, MUFA, PUFA: 28, 16, 5 E% HC CHO 61E%, FAT 16E%, PRO 23E% SFA, MUFA, PUFA: 5, 5, 3 E%	2	↔	LC ↑35 ± 21% (NS) HC ↓20 ± 9% (NS) Δliver TG with LC vs. HC *P* < 0.05	↔ glucose ↑ insulin in LC ↓ insulin in HC ↔ TG ↔ FA
[36]	Parallel n = 8 in LC (6M/2F) *n* = 9 in HC (7M/2F) 57 ± 8 and 58 ± 5 y (SD) BMI 28 ± 3 and 30 ± 2 Type 2 diabetes	7.4 ± 2.8 (SD) in LC 17.7 ± 9.7 in HC	LC CHO 40E%, FAT 42E% PRO 18E% SFA, MUFA, PUFA: 7, 27, 5E% HC CHO 53E%**,** FAT 28E%, PRO 19E% SFA, MUFA, PUFA: 6, 16, 4E%	8	↔	LC ↓ 25% vs. pre 7.4 ± 2.8% to 5.2 ± 2.7% (SD) HC ↔ vs. pre	LC vs. pre ↔ glucose ↔ insulin ↔ HOMA-IR ↓ HbA1c ↔ TG
[41]	Crossover *n* = 12 (6M/6F) 55 ± 14 y (SD) BMI 32.0 ± 4.2 HOMA-IR 4.3	11.2 ± 2.1%	LC CHO 34E%, FAT 44E%, protein 22E% SFA, MUFA, PUFA: 31, 51, 19% of total fat HC CHO 49E%, FAT 21E%, protein 30E% SFA, MUFA, PUFA: 36, 39, 24% of total fat	6	↔	LC ↓39 ± 4% vs. pre 14.2 ± 11.7% to 8.6 ± 7.0% HC ↔ vs. pre	LC vs. pre ↔ glucose ↓ insulin ↓ HOMA-IR ↑Δ GINF vs. HC HC ↔ glucose ↔ insulin ↔ HOMA-IR
[44]	Single-arm longitudinal *n* = 10 (4M/6F) 46 y (35–52) (IQR) BMI 31.1 (25.6–40.6) HOMA-IR 2.7 (2.0–4.8)	13% (median)	LC CHO 38E%, FAT 39E%, protein 23E% SFA, MUFA, PUFA 15, 15, 5E%	24	LC ↓ 1.9 kg vs. pre	LC ↓ 36 ± 26% (SD) vs. pre 13% to 5%	LC ↓ glucose ↓ insulin ↓ HOMA-IR ↔TG
[38]	Parallel, for *n = 7* crossover *n* = 13 (10M, 3F) 36 ± 3 y BMI 33.6 ± 1.3 Normal fasting glucose 2h OGTT glucose 5.2 ± 0.3 mmol/l	LC 8.3 ± 7.9% HC 9.4 ± 7.5%	LC CHO 27E%, FAT 55E%, PRO 18E% (SFA 25E%) HC CHO 62E%, FAT 20E%, PRO 18E% (8E% SFA) CON (pre-intervention) CHO 47E%, FAT 35E%, PRO 18E% (SFA 12E%)	4	↔	LC ↔ vs. CON HC ↓ 14% vs. CON (9.4 ± 7.5% to 7.2 ± 7.7%)	LC and HC vs. pre ↔ insulin ↔ TG ↔ FA
[43]	Crossover *n* = 11 NAFLD (M) 59 y (49–64) BMI 29 ± 0.3 HOMA2-IR 2.8 ± 0.1 *n* = 14 CON (M) 54 y (41–65) BMI 28 ± 0.5 HOMA2-IR 2.3 ± 0.3	NAFLD 17.2 ± 2.7% (7.9–36.8%) CON 2.5 ± 0.3% (0.5–4.6%)	LC CHO 42–44E%, FAT 33–34E%, protein 22–25E% SFA 13–14E% HC CHO 50–54E%, FAT 26–28E%, protein 18–24E% SFA 9–12E%	12	LC ↓ 2 kg vs. HC (both in NAFLD and CON)	NAFLD LC ↓41% vs. HC 14.2 ± 3.2% vs. 24.2 ± 6.8% CON LC ↓58% vs. HC 3.6 ± 1.3% vs. 1.5 ± 0.3%	LC vs. HC (both groups) ↔ glucose ↔ insulin ↔ HOMA2-IR ↔ TG ↓ FA (NAFLD)
[35]	Parallel *n* = 15 (8M/7F) in LC *n* = 15 (7M/8F) in HC *n* = 13 (10M/3F) in CON 62 ± 10, 63 ± 13, 60 ± 12 y (SD) BMI 30 ± 3, 31 ± 3, 32 ± 5 Prediabetes by ADA criteria	LC 9.8 ± 2.8% HC 6.9 ± 1.1% CON 11.2 ± 2.5%	LC CHO 36E%, FAT 46E%, PRO 18E% (SFA, MUFA, PUFA:11, 22, 5E%) HC CHO 54E%, FAT 28E%, PRO 18E% (SFA, MUFA, PUFA: 9, 7, 4E%) CON CHO 48E%, FAT 34E%, PRO 18E% (SFA, MUFA, PUFA: 12, 8, 4E%)	12	↔	LC ↓18 ± 3% vs. pre 9.8 ± 2.8% to 8.0 ± 2.5% HC ↔ vs. pre CON ↔ vs. pre	LC ↔ glucose ↔ insulin ↔ glucose tolerance ↔ TG
[40]	Single-arm longitudinal *n* = 10 (8M/2F) 54 ± 4 y BMI 34.1 ± 1.2 HOMA-IR 6.0 ± 1.4	16.0 ± 2.3%	LC CHO 4E%, FAT 72E%, PRO 24E% SFA, MUFA, PUFA: 17, 59, 23% of total fat 24E% protein	2	↓1.8 kg	↓39% vs. pre 16.0 ± 2.3% to 9.7 ± 1.9%	↔ glucose ↓ insulin ↓ HOMA-IR ↓ TG ↔ FA
[34]	Parallel LC *n* = 26 (11M/15F) HC *n* = 25 (11M/15F) 51 ± 13 and 53 ± 9 y (SD) BMI 31.5 ± 4.1 and 30.2 ± 5.6 HOMA-IR 3.7 ± 1.9 and 3.0 ± 1.0	LC 34.2 ± 16.3% HC 21.5 ± 10.0%	LC CHO 37E%, FAT 45% protein 19E% SFA 10E%, MUFA 55% of total fat HC CHO 48E% FAT 31E% protein 21E% SFA 10E%, MUFA 43% of total fat	12	LC ↓ 2 kg HC ↓ 1.7 kg	LC ↓30% vs. pre 34.2 ± 16.3% to 24.0 ± 14.7% HC ↓29% vs. pre 21.5 ± 10.0% to 15.3 ± 7.7%	LC ↔ glucose ↔ insulin ↔ HOMA-IR ↓HbA1c ↓ TG HC ↔ glucose ↔ insulin ↑ HOMA-IR ↔ HbA1c ↔ TG
[42]	Crossover *n* = 29 (20M/8F) 64 ± 8y (SD) BMI 30.1 ± 5.2 Type 2 diabetes	pre-LC 5.8% (2.1–12.5%) pre-HC 3.3% (1.4–8.7%) median (IQR)	LC CHO 30E%, FAT 40E%, PRO 30E% SFA, MUFA, PUFA: 13, 18, 8E% protein 30E% HC CHO 50E%, FAT 33E%, PRO 17E%	6	↔	LC ↓ 41% vs. pre 5.8% to 3.4%	LC vs. pre ↓ glucose ↔ insulin ↔ HOMA-IR ↓ HbA1c ↓postprandial glucose, insulin ↓ TG ↔ FA
[39]	Parallel *n* = 16 (7M/9F) in LC *n* = 16 (9M/7F) in HC 14 ± 2 and 15 ± 3 y (SD) BMI 35.9 ± 6.7 and 38.4 ± 7.3 HOMA-IR 7.5 ± 5 and 7.5 ± 6	LC 18.6 ± 9.0% HC 12.4 ± 9.6%	LC CHO 29E%, FAT 47E% protein 24E% SFA <10E% HC CHO 50E% FAT 53E% protein 19E%	8	LC ↓ 2 kg HC ↔	LC ↓32% vs. pre 18.6 ± 9.0% to 12.6 ± 6.6% HC ↔	LC ↔ glucose ↓ insulin ↓ HOMA-IR ↔ TG HC ↔ glucose ↑ insulin ↑ HOMA-IR ↔ TG

Dietary intervention studies manipulating carbohydrate and fat (or protein) intake in overweight or obese individuals with steatosis. Studies are listed according to their publication date. All interventions were designed as per eucaloric, although a minor weight loss was obtained in some studies. Intervention diets with reduced carbohydrate is referred to as LC, whereas diets with moderate to high-carbohydrate intake is referred to as control (CON) or HC according to the nomenclature used in the respective studies. In some studies, no information was provided on carbohydrate type in the LC diets, whereas in others, the carbohydrates were high-glycemic [36], wholegrain carbohydrates [41], low in sugars, including fructose [38,44], exchange of sugars for starch [43], or exclusion of refined carbohydrate, added sugar and fructose [39].

↑ and ↓: significant increase or decrease, respectively. ↔ no significant change. Data are means ± SEM unless otherwise noted.

Abbreviations: ADA, American Diabetes Association; CHO, carbohydrate; E%, energy%; F, female; FA, fatty acid; HC, high carbohydrate; LC, low carbohydrate; M, male; NAFLD, nonalcoholic fatty liver disease; OGTT, oral glucose tolerance test; PRO, protein; TG, triacylglycerol.

## Carbohydrate reduction in individuals with steatosis

In individuals with steatosis and indices of insulin resistance, a beneficial effect of carbohydrate replacement was apparent on hepatic TG content during eucaloric conditions. Thus, when 6–12 wk of reduced carbohydrate diets (29–42E% carbohydrate, 40–47E% fat, and 18–30E% protein) were provided to obese female and male adolescents [39] or obese men and women [34–36,41–43] with average liver TG of 6–34% and either elevated HOMA-IR index (average between 2.7 and 7.5 index values), prediabetes, or type 2 diabetes, liver TG content was reduced by 18–41% compared with pre-intervention or with conventional diet (Table 1). In one study, a similar reduction in liver TG (−29% compared with −30%) was obtained in each parallel intervention arm with consumption of 48E% carbohydrate and 31E% fat, as with 37E% carbohydrate and 45E% fat (Table 1, [34]), potentially ascribed to the moderate carbohydrate intake of both groups.

Thus, in these 7 studies of both male and female individuals (*n* = 105 in total) with hepatic steatosis and insulin resistance, several weeks of moderate carbohydrate reduction (intake of ∼30–40E%, corresponding to ∼190–250 g/d) were sufficient to reduce hepatic TG content by 25–41%, under conditions of increased fat intake (40–47E%, primarily unsaturated fatty acids) and increased protein (Table 1). The habitual carbohydrate intake was reported to be 38–46E% (195–333 g/d), with an average energy intake of 7.9–12.8 MJ. Although underreporting for energy and macronutrient intake may be possible, this suggests that significant effects were obtained with only 10–15E% carbohydrate reduction. One shorter term study of 2 wk duration provided a diet with only 4E% carbohydrate (31 ± 1 g/d), 72E% fat, and 26E% protein, and obtained a marked decrease in hepatic TG content of 44% compared with pre-intervention [40]. Severe carbohydrate restriction thus has the potential to reduce hepatic TG content within the short term. In 5 of the studies, a minor average weight loss of −1.7 to −2kg was present during the 2–12 wk of interventions [34,39,40,43], despite energy provision aiming at eucaloric requirements. This could make a small contribution to the findings.

Besides total content, the type and structure of the carbohydrates (, that is, monomeric isoform, extent of polymerization, and degree of α- and β-linkage), as well as solid or liquid form, all determine the glycemic response and thus the insulin secretion, with subsequent effects on hepatic metabolism. Simple sugars and fructose have been speculated to be detrimental to liver health [45,46]. In the reduced carbohydrate interventions in Table 1, some studies manipulated with added sugar, glycemic index, and fructose content, whereas others did not report on any alterations in carbohydrate type (see legend of Table 1). One could hypothesize that sugar and fructose intake is less detrimental when energy and carbohydrate excess are not apparent [47,48]. This remains to be established in well-controlled studies with accurately matched total carbohydrate intake.

Two intervention studies observed no significant effect on hepatic TG content when providing diets with higher total and saturated fat content (55–56E% total fat and 25–28E% SFA) [37,38] (Table 1). In obese women with HOMA-IR of 2.6 and 10% hepatic TG, a reduction from the habitual carbohydrate intake of 45E–31E% (with 56E% fat) for 2 wk led to a (nonsignificant) increase of 35% in hepatic TG content compared with pre-intervention [37]. Also, when compared with a pre- intervention diet comprising 47E% carbohydrate, hepatic TG content remained unchanged (8% compared with 7%) in obese men and women with normal glucose tolerance after intake of 27E% carbohydrate and 55E% fat for 4 wk [38]. Specific to these studies was the provision of a higher saturated fat content than in the above-mentioned studies, in which increased monounsaturated fat was provided. Moreover, total dietary fat content was higher than in the studies providing 40–47E% fat (where liver TG was reduced), but still lower than the 72E% fat provided in the study by Mardinoglu et al. [40] who also found reduced liver TG. Finally, the discrepant findings could also relate to a diminished effect of the moderate carbohydrate reduction (to 27E% and 31E%) in individuals with normal glucose metabolism.

When it comes to the role of dietary FA saturation in regulation of liver TG content, one study investigated the effect of FA type in obese individuals and did over 10 wk report, a small 9% increase in hepatic TG content (from 3.2% to 3.5%) with a 5E% increase in saturated fat intake and a 28% decrease in hepatic TG content after a 9E% increase in n-6 polyunsaturated fat intake, with similar energy and fat intake between groups [49]. Further studies should establish whether mono- and polyunsaturated fat have beneficial effects on the regulation of TG content. Considering that an excessive intake of saturated fat may potentially be of concern for hepatic TG, and that we have several observations of positive outcomes for hepatic TG content obtained with a high amount of monounsaturated fat in the diet, it seems reasonable to conclude that a high dietary amount of saturated fat may not be favorable for liver TG when compared with unsaturated fat.

Besides an upregulation of fat intake to compensate for the reduced dietary carbohydrate, protein intakes were 18–30E% in the above-mentioned studies, why a concomitant replacement by increased protein may also play a role. One study provided a higher protein diet for 6 wk, with protein intake elevated from the habitual 17E to 30E% (protein replaced fat in the diet) whereas carbohydrate intake remained at ∼40E%) in obese individuals with steatosis (hepatic TG of 15%) and type 2 diabetes, and found a reduction in liver TG of 48 and 36% with animal and plant protein, respectively [50]. Although these findings were obtained with a reduction in BMI of −0.8 and −0.5 kg·m^−2^, it could suggest that increased protein intake could also play an additive role in reducing TG accumulation in the liver.

## Carbohydrate reduction in individuals without steatosis

The effect of eucaloric carbohydrate replacement diets on hepatic TG content has also been investigated in individuals without steatosis [43,[51], [52], [53], [54]]. Umpleby et al. [43] included, in parallel to their study in individuals with steatosis, an intervention arm with nonsteatotic overweight individuals with insulin resistance, that is, an elevated HOMA2-IR index [43]. After ingestion of a 12 wk diet with either 44E% carbohydrate (34E% fat) and 54E% carbohydrate (26E% fat) in a crossover design, liver TG was 58% lower after the low (1.5 ± 0.3%) compared with higher carbohydrate diet (3.6 ± 1.3%) [43]. However, in normoglycemic normal weight or overweight women and men with an average liver TG content below 4%, no significant changes in hepatic TG content were found when ^1^H-MRS was applied after 3, 4, or 12 wk of the diet with 34–38E% carbohydrate and 43–49E% fat [[51], [52], [53]], when compared with a control diet or pre-intervention. The contrasting findings of lower liver TG content after moderate (44E%) compared with higher (54E%) carbohydrate intake in Umpleby et al. [43], could relate either to the presence of insulin resistance in these individuals or the unsaturated profile of the high-fat diet.

## Concomitant Effect of Reduced Dietary Carbohydrate on Indices of Insulin Resistance

In the studies where diets with reduced carbohydrate (4E%, or 29–40E%), increased fat (72E%, or 39–47E%), and increased protein (up to 30E%) were provided to individuals with steatosis and insulin resistance or type 2 diabetes, 3 studies evaluated HbA1c and all could demonstrate improved glycemic control (average reduction of −0.2 to −0.6% in HbA1c) within the observation period of 6–12 wk [34,36,42]. The HOMA-IR index was assessed in 8 studies, 4 reported a lowering of the HOMA-IR index, due to a 14–43% reduction in fasting plasma insulin [[39], [40], [41],^44^], whereas the HOMA-IR index was unchanged in the other 4. Elevated fasting insulin concentrations seem to be a hallmark in hepatic steatosis, likely associated with the inverse association between hepatic TG content and insulin clearance by the liver [9]. None of the studies evaluated fasting plasma C-peptide, then why the effect on beta-cell secretion remains to be investigated. It could also be speculated that increased hepatic insulin clearance after reduced carbohydrate regimens could represent a mechanism for the reduced circulating insulin, because the absolute carbohydrate intake seems important in the regulation of insulin clearance, as reviewed in Bojsen-Møller et al. [9]. In the postprandial periods, less insulin secretion because of lower carbohydrate intake will reduce insulin availability to the liver, and with a potential contribution of increased hepatic insulin clearance, this would lead to lower systemic insulin levels. Lower insulin exposure (both hepatic and systemic) will have subsequent effects on liver and whole-body substrate oxidation and metabolism, in turn, influencing FA availability to and within the liver.

Overall, reduced carbohydrate and increased fat intake can under eucaloric conditions reduce liver TG content as well as improve whole-body glucose metabolism in individuals with steatosis and indices of insulin resistance.

## Potential Mechanisms by which Reduced Carbohydrate Intake Can Lower Hepatic TG Content

The regulation of hepatic TG content results from a complex interplay between FA availability to the liver (FAs from chylomicron remnants and spillover from peripheral chylomicron-TG hydrolysis, and FAs from adipose tissue lipolysis), and FA metabolism within the liver (FA oxidation, FA synthesis from DNL, FA esterification, TG lipolysis, and TG secretion within lipoproteins). In the following, it will be reviewed which of these aspects of hepatic FA metabolism a reduced carbohydrate and increased fat intake will modify.

## FA availability to the liver

An increase in fat intake when fat is replacing, carbohydrate in eucaloric diets leads to increased postprandial plasma chylomicron-TG concentrations and hence a greater chylomicron-derived plasma FA availability. The apparent controversy to the concomitant reduction in hepatic TG with such regimens is likely to be explained by the lymphatic chylomicron absorption route. Hence, a significant peripheral clearance into adipose tissue and skeletal muscle of up to 80–90% of chylomicron-TG is obtained in healthy individuals [55]. Under such conditions, a minor fraction of dietary FAs from chylomicron remnants or spillover FAs from peripheral chylomicron-TG hydrolysis are available for the liver. Thus, diet-derived FAs do not represent the major source of TG stored in the liver, as demonstrated by Donnelly et al. [56] who found in individuals with steatosis that only 15% of liver TG originated from dietary fat obtained after 4 d of tracer administration, whereas eating a diet with 55E% carbohydrate and 30E% fat [56]. This is supported by tracer studies of postprandial plasma VLDL-TG, because the FA sources of circulating VLDL-TG reflect the FA sources of intrahepatic TG [56]. Only 13–29% of plasma VLDL-TG was comprised of FAs from chylomicron remnants and chylomicron-TG hydrolysis spillover in the postprandial state after enteral or oral administration of TG and FA tracers in normal-weight healthy women and men [57,58]. Although the contribution of diet-derived FAs to liver TG has not been assessed during high-fat intake, these studies suggest a relatively small role of diet-derived FAs in liver TG accumulation.

**FIGURE 1:**
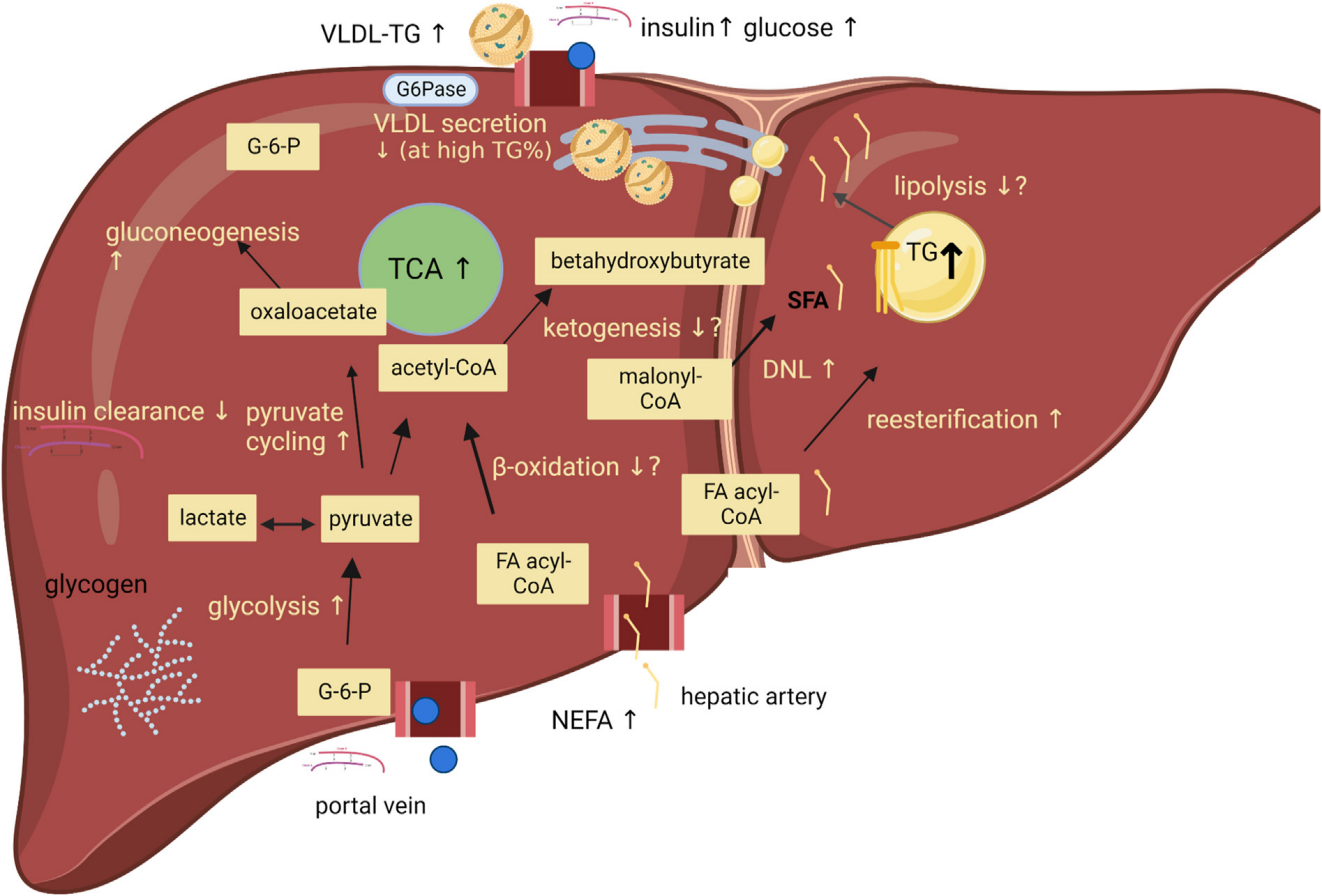
Illustration of the lipid and glucose metabolic pathways that are suggested to be altered in liver steatosis. Adipose-derived plasma nonesterified fatty acids (NEFA or FA) in the circulation is found to be elevated in individuals with steatosis, and appears to make a significant contribution to triacylglycerol (TG) accumulation in the liver. Splanchnic (hepatic) FA oxidation has not been compared in individuals with steatosis vs. normal liver fat, and divergent findings in studies measuring beta-hydroxybutyrate production rate or plasma concentrations in individuals with steatosis vs. normal liver fat also limit conclusions on whether FA oxidation or ketogenesis is lower when steatosis is present. Along with an increased gluconeogenesis, a hallmark of the steatotic and insulin-resistant liver, it has been found that hepatic mitochondrial oxidative metabolism and the activity of the tricarboxylic acid (TCA) cycle are elevated in individuals with steatosis, along with indices of elevated glycolysis and pyruvate cycling in the liver. This seems to be coupled with a well-documented increase in de novo lipogenesis (DNL) in the steatotic state. G-6-P, glucose-6-phosphate; G-6-Pase, glucose-6-phosphatase; VLDL-TG, very low-density lipoprotein-triacylglycerol. Created with Biorender.com.

Adipose-derived plasma FAs appear to make a significant contribution to TG in the liver. In fasting lean men and women, 77% of VLDL-TG represented adipose-derived plasma FAs, whereas this amounted to 44% in the fed state [56,57,59]. In obese individuals matched for body fat percentage, fasting plasma FA concentration and systemic palmitate rate of appearance (Ra) measured by palmitate tracer infusion were 30 and 40% higher, respectively, in individuals with hepatic steatosis compared with individuals with normal liver TG content [60]. Moreover, in obese men and women with a wide range of hepatic TG content (1–46%), hepatic TG content correlated inversely with suppression of systemic Ra of palmitate during low-dose insulin infusion (plasma insulin ∼48 μU/ml) [61]. Increased plasma FA concentrations, both in the fasting state and after intake of a meal, potentially due to adipose tissue insulin resistance at the level of lipolysis, could thus contribute to liver TG accumulation (Figure 1). Concomitantly with these associations between greater circulating FA availability and TG accumulation in the liver, an increased protein content of the FA transporter cluster of differentiation 36, which is important for FA uptake, has been obtained in liver samples of individuals with steatosis compared with normal liver fat [62]. Endogenous plasma FAs thus represent an important source of TG in liver steatosis.

Reduced carbohydrate and increased fat regimens appear to have a minor effect on fasting plasma FA concentration and plasma FA Ra in obese individuals with steatosis. Fasting plasma FA concentrations did not change compared with pre-intervention after 2–6 wk interventions with 4E% or 27–31E% carbohydrate and 72E% or 40–56E% fat in overweight or obese individuals with steatosis [37,38,40,42], although one study showed that fasting plasma FA concentration, as well as fasting plasma FA Ra, were 17% and 13% lower, respectively, after 12 wk of 43E% carbohydrate and 34E% fat compared with after a 12 wk diet with 54E% carbohydrate and 26E% fat [43]. The lower plasma FA Ra was obtained concomitantly with unchanged fasting plasma insulin, suggesting insulin-independent suppression of lipolysis.

In normoglycemic normal to overweight individuals without known steatosis, reduced carbohydrate regimens seem to increase adipose lipolysis. Thus, adipose tissue interstitial glycerol concentration of overweight men was 17% greater after 2 wk of eucaloric intake of 9E% carbohydrate and 54E% fat compared with a diet comprising 43E% carbohydrate and 32E% fat. This was associated with 57% lower fasting plasma insulin after the diet comprising 9% carbohydrate [63]. Moreover, fasting plasma FA Ra was 30% higher after 2 wk on a 30E% carbohydrate and 55E% fat diet compared with 75E% carbohydrate and 10E% fat in normoglycemic normal-weight men and women, concomitantly with a nonsignificant 10% lower fasting insulin [64]. This suggests less availability of insulin to inhibit lipolysis during conditions of low carbohydrate intake. Hence, eucaloric lowering of carbohydrate intake from 60E% to 5E% (60E% fat) or 20E% (64E% fat) was, in normoglycemic nonobese individuals, associated with an increase in fasting plasma FA concentration after a few days [65] or several weeks [66]. This was obtained concomitantly with a 50% lower 24-h plasma insulin concentration and 24–43% lower fasting insulin concentrations [63,66]. These indices of increased adipose lipolysis in nonobese may relate to an intact adipose tissue sensitivity to the reduced systemic insulin levels with low carbohydrate intake.

The availability of adipose-derived plasma FAs to the liver does thus not seem to be lower with carbohydrate reduction in obese individuals with steatosis, and may even be increased in nonobese individuals, why this does not seem to be a factor explaining the lowering of hepatic TG. Considered together with the increased availability of chylomicron-derived FAs in fat-substituted diets, low carbohydrate regimens likely lead to reduced liver TG via regulation of FA metabolism within the liver.

## FA Metabolism within the Liver

### Hepatic beta-oxidation and ketogenesis

Hepatic beta-oxidation and ketogenesis represent pathways for FA disposal by the liver. The hepatic FA utilization rate in healthy human individuals, determined from the arterial-hepatic venous gaseous exchange and FA uptake correlated positively with the production of the ketone bodies acetoacetate and beta-hydroxybutyrate in the fasted state [67]. Similarly, the plasma concentration of beta-hydroxybutyrate was also highly correlated with hepatic FA oxidation when determined by ^11^C-palmitate administration combined with PET-CT liver imaging of obese individuals [68]. This has led to a frequent use of systemic beta-hydroxybutyrate concentration as a surrogate marker of hepatic beta-oxidation. As ketogenesis and beta-oxidation may not correlate under all physiological conditions, hepatic acetyl-CoA content may be a better predictor of ketogenesis rate, as shown by noninvasive nuclear magnetic resonance (NMR) spectroscopy in rodent liver [69].

It has been questioned whether liver ketogenesis is impaired when steatosis is present. When the beta-hydroxybutyrate production rate was measured in the fasting state by co-infusion of labeled acetate and beta-hydroxybutyrate, one study showed lower [70] whereas another showed a similar [71] beta-hydroxybutyrate production rate in obese individuals with steatosis compared with controls with normal liver TG content. Measurements of fasting plasma beta-hydroxybutyrate concentration in obese individuals with steatosis have also revealed mixed findings, because 3 studies found fasting beta-hydroxybutyrate to be 16–38% lower [[72], [73], [74]], whereas others reported similar [75,76] or higher [77] levels when compared with individuals with normal liver TG. These discrepant findings do not enable solid conclusions as to whether ketogenesis is reduced when steatosis is present. Lack of prior dietary control of carbohydrate and fat intake may have contributed to the divergent findings.

**FIGURE 2:**
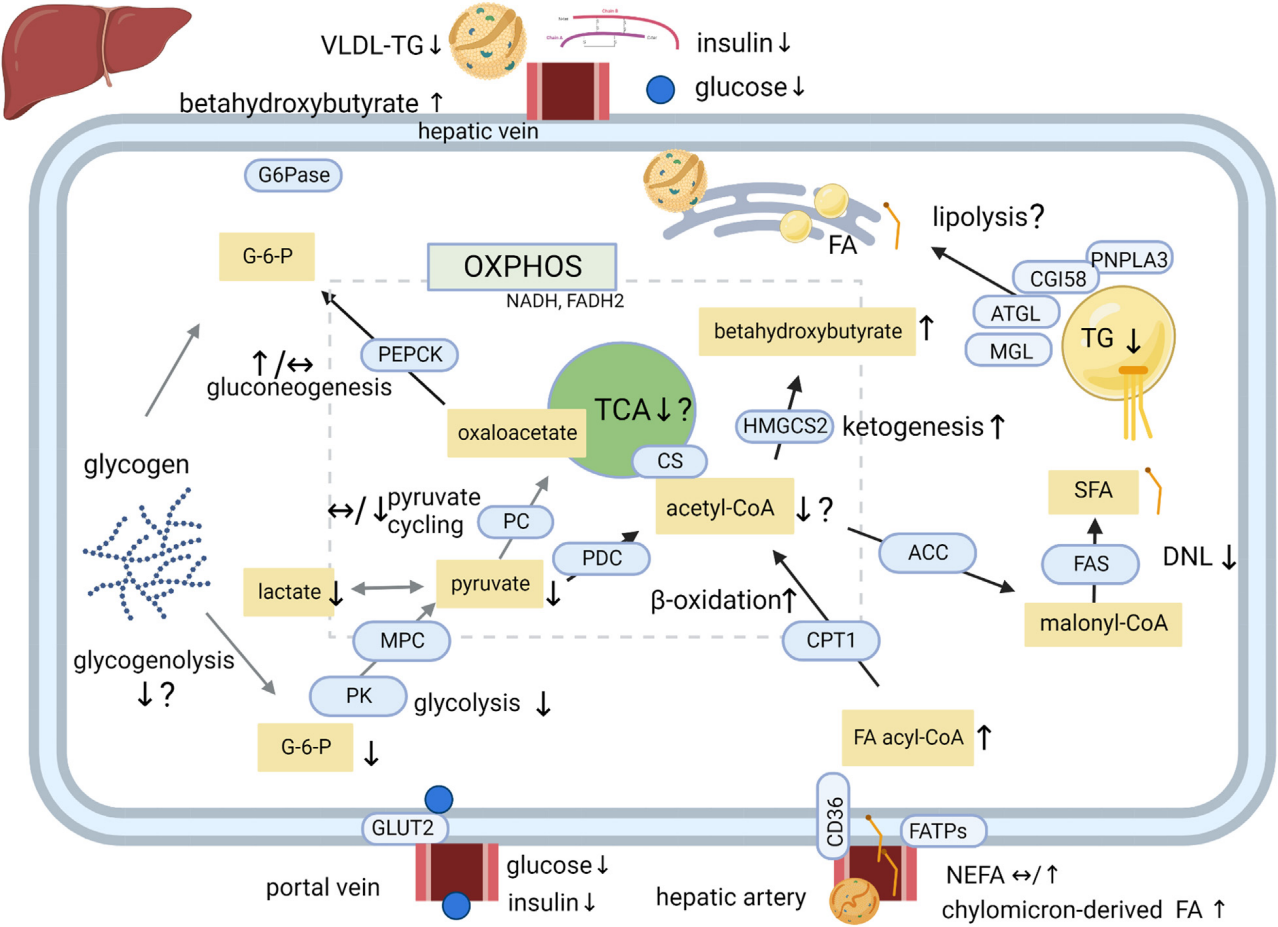
Pathways by which reduced carbohydrate intake could modify fatty acid (FA) and glucose metabolism in the liver. With intake of a fat-replaced reduced carbohydrate diet, glucose availability in the portal vein is lowered, whereas the availability of chylomicron-derived FAs in the mesenteric lymph is increased. Reduced hepatic glucose availability may, in turn, lead to reduced glycolysis, and hence lower pyruvate availability for acetyl-CoA production via the pyruvate dehydrogenase complex (PDC), and also lower pyruvate cycling to oxaloacetate via pyruvate carboxylase. Reduced carbohydrate intake is shown to reduce liver TCA cycle flux [129]. Lower glucose availability could potentially reduce liver glycogen content (which awaits to be assessed), but it is known that eucaloric reduced carbohydrate intake leads to reduced fasting liver glycogenolysis [79,80]. Gluconeogenesis is shown to be either increased [80] or unchanged [79] with reduced carbohydrate and increased fat intake. A reduced hepatic glucose availability concomitant with increased availability of chylomicron-derived FAs would likely increase the fraction of acetyl-CoA that is derived from beta-oxidation, and splanchnic FA oxidation is shown to be increased with reduced carbohydrate intake [64]. An increased hepatic beta-oxidation is likely stimulating increased ketogenesis [30,40]. The concomitant reduction in glycolysis-derived acetyl-CoA could be speculated to reduce total levels of acetyl-CoA, and hence reduce the flux via acetyl-CoA carboxylase and lower malonyl-CoA availability for fatty acid synthase. This could be mechanisms in the reduced de novo lipogenesis (DNL) [40,95]. ATGL, adipose triglyceride lipase; CD36, cluster of differentiation 36; CGI-58, comparative gene identification 58; CPT1, carnitine palmitoyl transferase 1; CS, citrate synthase; FATPs, fatty acid transport proteins; G-6-Pase, glucose-6-phosphatase; HMGCS2, 3-hydroxy-3-methylglutaryl-CoA synthase 2; MGL, monoacylglycerol lipase; MPC, mitochondrial pyruvate carrier; OXPHOS, oxidative phosphorylation; PEPCK, phosphoenolpyruvate carboxykinase; PK, pyruvate kinase; PNPLA3, patatin-like phospholipase domain-containing protein 3; VLDL-TG, very low-density lipoprotein-triacylglycerol. Created with biorender.com.

It is, however, well documented that hepatic FA oxidation as well as fasting plasma ketones increase in response to reduced carbohydrate high-fat diets, also at eucaloric conditions. Splanchnic FA oxidation, measured by FA tracer and subsequent arterial-hepatic venous labeled CO_2_ appearance, was 81% higher after 2 wk intake of 30E% carbohydrate and 55E% fat than after the intake of 75E% carbohydrate and 10E% fat in lean individuals [64], suggesting a pronounced effect on hepatic FA oxidation. In obese individuals with steatosis, eucaloric reduced carbohydrate intake (4 and 8E%) and 72 and 58E% fat for 2 wk increased the fasting plasma beta-hydroxybutyrate concentration and total ketones by 150% compared with pre-intervention [30,40], with the increase in ketones correlating with the reduction in hepatic TG content [30]. Also in lean and overweight individuals (in which liver TG content was not assessed), eucaloric reduced carbohydrate diets (5–20E%) with 60–64E% fat led to an increase in the fasting plasma beta-hydroxybutyrate concentration by 150–400% after either 3 d, 1 wk, or 6 wk when compared with pre-intervention or a control diet [65,66,78]. Thus, a eucaloric reduction in carbohydrate intake to 20E% or less (∼30–130 g) and an increased fat intake to 60E% or more leads to increased ketone production, indicating increased hepatic beta-oxidation (Figure 2). The stimuli to increase beta-oxidation could be substrate availability, along with altered signaling or transcriptional mechanisms in the liver.

It is known that FAs act as signaling molecules and can increase the transcription of genes involved in FA metabolism [81]. A potential mechanism in the upregulated beta-oxidation after a diet with reduced carbohydrates and increased fat [82] could be the activation of hepatic peroxisome-proliferator activated receptors (PPARs) by FAs. In support of a central role of increasing hepatic FA oxidative capacity is the apparent efficacy of pharmacological PPAR ligands in the treatment of NAFLD [83]. To this end, analysis of liver biopsies demonstrated a negative correlation between hepatic PPARα mRNA content and the degree of steatosis in 125 individuals with a broad range of BMIs [84]. In addition, hepatic RNA sequencing from obese individuals with steatosis revealed lower expression of PPARα, carnitine palmitoyl transferase 1 (CPT1), and also the gene encoding hydroxy-methylglutaryl-CoA synthase 2 (HMGCS2), the rate-limiting enzyme in ketogenesis [85]. In the liver of obese individuals with NASH, long-chain acylcarnitine content was elevated compared with control [86], indicating insufficient beta-oxidation. Moreover, maximal beta-HAD activity was found to be lower in liver samples of obese individuals with NAFLD compared with normal liver fat [87]. These findings highlight a low capacity for FA oxidation, and also ketone synthesis, in the steatotic liver, indicating the potential of activating PPAR receptors by dietary changes.

### Hepatic DNL

Hepatic DNL can be estimated by labeled acetate infusion (and subsequent FA synthesis from acetyl-CoA) or by intake of deuterated water (and subsequent incorporation of labeled hydrogen in the FA acyl chain), whereby the occurrence of the palmitic acid end-product of DNL in the liver(-derived) TG is measured. In individuals with steatosis, DNL-generated palmitic acid comprised 11–26% of the FAs enclosed in plasma VLDL-TG [56,[86], [87], [88], [89], [90]] compared with 1–10% in lean or obese individuals with normal liver TG [88,[90], [91], [92]], when investigated in either fasted or fed state. Two studies provided deuterated water for a period of 2–5 wk, and obtained a VLDL-TG contribution from DNL of 11% and 18% in lean nonsteatotic individuals, and 38% and 43% in obese individuals with steatosis [93,94]. The rate of DNL seems thus to be increased in the steatotic liver.

Downregulation of DNL in response to reduced carbohydrate and increased fat intake has been demonstrated under eucaloric conditions. With marked carbohydrate restriction to 4E% (72E% fat) in the study by Mardinoglu et al. [40], deuterated water administration showed that DNL-derived FAs in plasma VLDL-TG was 75% reduced within 3 d. Also with less pronounced carbohydrate reduction, deuterated water revealed 25% lower DNL after 3 d of 48E% carbohydrate and 37E% fat compared with intake of 59E% carbohydrate and 23E% fat in both obese normoglycemic and obese T2D individuals [95]. Finally in obese individuals, acetate-estimated DNL comprised 18% of plasma VLDL-TG in the fasting state compared with 33% after 2 wk of 55E% carbohydrate and 30E% fat intake and 75E% carbohydrate and 10E% fat intake, respectively [96]. Accordingly, even moderate reductions in carbohydrate intake may reduce hepatic DNL and thereby contribute to reduce TG accumulation (Figure 2). A lower prehepatic insulin concentration with reduced carbohydrate could play a role for insulin stimulation of DNL, and the lower DNL after intake of 55E% carbohydrate in the study by Hudgins et al. [96] was hence associated with 45% lower 24h plasma insulin levels at the end of intervention.

The end-product of DNL is the saturated FA palmitic acid. In overweight men and women with liver TG content ranging from normal to marked steatosis, DNL, determined by deuterated water, was positively correlated with the fraction of saturated FAs measured in liver biopsy samples [97]. In the study by Mardinoglu et al. [40], the reduction in DNL correlated with the reduction in hepatic TG content in obese individuals with steatosis, and the change in DNL was associated with a decreased mol% of saturated FAs in the liver, assessed by MRS. The mechanisms by which a more desaturated FA profile of liver TG after reduced carbohydrate and increased (unsaturated) fat regimens could influence liver FA metabolism (for example, FA oxidation, FA esterification or TG lipolysis), await to be investigated.

### Hepatic lipolysis

The hepatocellular pool of TG must undergo lipolysis before FAs can be liberated and enter beta-oxidation, TG (VLDL-TG) synthesis, synthesis of phospholipids or serve as signaling molecules. Adipose triglyceride lipase (ATGL, also known as PNPLA2) is the primary lipase catalyzing hydrolysis of liver TG, as hormone sensitive lipase (HSL) does not seem to be expressed in human liver cells [98]. Monoacylglycerol lipase (MAGL) catalyzes the last step in TG hydrolysis. Reduced hepatic lipolytic activity may be a potential mechanism in steatosis [17], although sparse evidence on lipolytic regulation in the liver is available from human studies. Although ATGL mRNA is similar in liver samples from individuals with and without steatosis [99], ATGL protein content has not been investigated. However, immunohistochemistry of the human liver revealed lower MAGL protein expression in specimens from fibrotic compared with normal liver [100].

Not much is known about the role of dietary carbohydrates and fat availability in hepatic lipolysis. It is well known that insulin is a negative regulator of lipolysis in adipose tissue and that adipose tissue lipolysis is higher after eucaloric reduced carbohydrate high-fat diets [63,64]. In contrast, the regulation of hepatic lipolysis by insulin has been less studied. Insulin signaling via protein kinase B (AKT) and a subsequent decrease in cAMP is one mechanism for inhibition of lipolysis, while AKT-mediated inhibition of forkhead box O (FOXO) proteins and hence reduced transcription of ATGL represents another mechanism to regulate hepatic lipolysis, as shown in mice and hepatocytes [101]. Besides this, lipase activity is regulated by several proteins and co-activators, and here a link between lipolysis and steatosis is evident from genome-wide association studies. Patatin-like phospholipase domain-containing protein 3 (PNPLA3) is, as ATGL/PNPLA2, a member of the Patatin family of lipases. PNPLA3 is involved in lipolysis by interacting with and sequestering the co-activator comparative gene identification-58 (CGI-58), thus limiting lipid droplet access by ATGL, as shown in mice and cells [102]. It is well known that a common mutation (I148M) in the PNPLA3 gene is highly related to liver steatosis [103], and findings in vitro and in mice have shown that the I148M mutation results in increased PNPLA3 binding to CGI-58 and diminished ATGL-mediated lipolysis of hepatic TG [104].

Rodent studies suggest that carbohydrate feeding induces PNPLA3 in the liver. Hence, feeding mice high-carbohydrate or high-sugar diets led to elevated PNPLA3 protein in the liver compared with standard unpurified diet-fed mice [105]. The results of a human dietary intervention confirm a potential role of carbohydrate availability in the regulation of PNPLA3. In obese individuals with hepatic steatosis, divided into 2 groups on the basis of PNPLA3 genotype, 6 d of a hypocaloric low carbohydrate diet (<20 g/d) led to a larger decrease in hepatic TG content (-45%) in individuals with the 148M mutation than in individuals without (-18%) [106]. Individuals with the PNPLA3 gene variant, and thus increased susceptibility to diminished TG lipolysis in the liver, may therefore be more responsive to reduced dietary carbohydrate. It should however be noted that increased TG accumulation with the PNPLA3 I148M genotype may be less linked to hepatic insulin resistance than increased hepatic TG in noncarriers [107].

Reduced carbohydrate availability may therefore lead to less PNPLA3-inhibition of ATGL activity and thus increased lipolysis. Taken together, a high-carbohydrate intake may downregulate lipolysis in the liver, likely through a plethora of mechanisms but including downregulation of lipase expression and via induction of PNPLA3.

### Hepatic VLDL-TG secretion

Formation and secretion of VLDL lipoproteins from the liver to the circulation is another regulatory step determining the degree of hepatic TG accumulation. The VLDL-TG secretion rate in the fasting state [60,108] and after insulin infusion [108] is higher in obese individuals with steatosis (average TG of 11–23%) than in nonsteatotic individuals, and there is a positive correlation between VLDL-TG [60] or VLDL_1_-TG [108] secretion rate and hepatic TG content. However, this association seems to be abolished at liver TG contents above 10% [60,108]. The lack of a compensatory increase in VLDL-TG secretion in response to increasing liver TG formation has been ascribed to hepatic insulin resistance and regulation at the step of VLDL assembly. In dietary studies, applying ^13^C_3_- or ^125^I-labeling of VLDL-TG or ^14^C labeling of VLDL–TGFA, a 50–60% lower fasting VLDL-TG secretion rate was shown in lean and overweight men and women after 1–2 wk of eucaloric intake of 30–45E% carbohydrate and 40–55E% fat compared with carbohydrate-rich low-fat diets comprising 75–80E% carbohydrate [64,109,110]. These findings were associated with the regulation of the concentration of TG in VLDL particles, rather than changes in apoB secretion rate [110]. The reduced secretion rate after reduced carbohydrate and increased fat intake is likely reflective of a reduced hepatic TG accumulation under these dietary conditions. The effect of reduced carbohydrate and increased fat regimens on VLDL-TG kinetics in obese individuals with steatosis remains to be assessed.

## The role of dietary carbohydrate and fat in transcriptional regulation of hepatic substrate metabolism

During reduced carbohydrate and high-fat regimens, nutrient sensing in the liver will involve a reduced monosaccharide availability, combined with exposure to lower pancreatic insulin secretion and elevated FA availability. Summarizing the studies, it appears that the combination of reduced carbohydrate and increased fat intake (in some studies together with increased protein) lead to increased FA beta-oxidation and ketogenesis, while reducing DNL and glycolysis. Such changes likely result from the altered transcription of metabolic genes. Carbohydrate response element binding protein (ChREBP) is proposed as a regulatory point between carbohydrate sensing and lipid synthesis. ChREBP is a transcription factor that suppresses lipid metabolic genes, while increasing the transcription of genes in DNL and glycolysis [111]. ChREBP does in synergy with sterol regulatory element binding protein 1 (SREBP1c) coordinate the expression of DNL and lipogenic (esterification) enzymes. Hepatic ChREBP is activated by glucose and its metabolites, such as glucose-6-phosphate (G6P), as well as fructose [112] while SREBP1c is positively regulated by insulin. The activity of both ChREBP and SREBP1c is inhibited by unsaturated FAs [113,114]. ChREBPβ mRNA in the liver is higher in obese compared with lean individuals [115], and SREBP1c protein content is increased in the liver of human individuals with NAFLD compared with normal liver fat [116]. Attenuated ChREBP and SREBP1c activity with low carbohydrate diets may hence downregulate the molecular potential for DNL and glycolysis, while increasing the potential for FA oxidation and ketogenesis.

Hepatic FAs also bind to PPARs, such as PPARα [117], which, in turn, would increase the transcriptional abundance of lipid oxidative and ketogenic genes. PPARα gene expression is shown to inversely correlate with the degree of hepatic steatosis in individuals with NAFLD [84]. Increased FA availability may hence directly induce genes in FA utilization. As lipolysis is important to liberate FA ligands for PPAR signaling [118], this indicates that lipolytic activity in the liver is important in regulating FA oxidative genes.

## Dietary Carbohydrate Reduction and Mitochondrial Substrate Availability

Human studies reveal an increased oxidative metabolism in the liver of obese individuals with steatosis compared with lean individuals. Thus, fasting arterial-hepatic venous oxygen uptake was 61% higher in obese compared with lean men [119], and by use of ^13^C-proprionate administration combined with NMR spectroscopy, oxidation in the tricarboxylic acid (TCA) cycle was 60–100% higher in obese individuals with steatosis (an average TG content of 17% in both studies) compared with overweight or obese controls with normal liver fat [70,71]. Also, when measured ex vivo in liver tissue samples and isolated liver mitochondria by respirometry, higher respiration was obtained with TCA cycle substrates in obese individuals with steatosis (an average TG content of 27%) compared with lean [120]. This suggests an increased activity of the TCA cycle when steatosis is present, speculated to function as a progenitor of increased gluconeogenesis, a hallmark of the insulin-resistant steatotic liver (Figure 1). Also the conversion of pyruvate to the TCA cycle intermediate oxaloacetate (anaplerosis) was shown to be 30–50% higher in the obese individuals with steatosis [70,71]. One study did, however, not show a difference in liver mitochondrial TCA cycle flux between slightly overweight individuals with steatosis (an average TG content of 8%) and lean controls, when evaluated by labeled acetate and lactate infusion combined with ^13^C-MRS [121]. The different observations may relate to the different methodologies or differences in the degree of steatosis.

It could be speculated whether hepatic acetyl-CoA excess is present in the steatotic liver. Acetyl-CoA is produced either by oxidative decarboxylation of pyruvate from glycolysis, by beta-oxidation, or by oxidative degradation of amino acids. In the healthy liver, excess acetyl-CoA is disposed of by ketogenesis, as substrate in DNL or buffered as acetyl-carnitine. Acetyl-CoA also functions as an effector in gluconeogenesis. In the steatotic liver, the capacity for ketogenesis and also the carnitine availability for FA oxidation and acetyl-CoA buffering may be insufficient [122], whereas pathways such as DNL and gluconeogenesis are increased.

Several lines of evidence point to the role of increased pyruvate availability and increased hepatic glycolytic activity for the accumulation of liver TG. Hence, higher fasting plasma concentrations of pyruvate and lactate are reported in individuals with elevated compared with normal liver TG [70,123]. Also, a higher incidence of steatosis is evident in individuals with a gene variant leading to overexpression of the glycolytic enzyme liver glucokinase [124]. Moreover, increased gene expression of pyruvate kinase, catalyzing the final step in glycolysis, has been shown in liver biopsies from men with steatosis compared with normal liver fat [125]. Findings from a metabolomics study of arterial-hepatic venous blood confirmed correlations between the degree of liver fat and the following: liver pyruvate transport, TCA cycle oxidation, and pyruvate to oxaloacetate conversion in individuals with normal to high liver TG [123]. Accumulation of glycolytic products could thus lead to higher acetyl-CoA production, which represents a substrate for DNL and for glycerol synthesis, and thereby, in turn, could contribute to TG accumulation.

In vitro and rodent studies have shown that pharmacological inhibition of hepatic mitochondrial pyruvate entry could be beneficial for reducing TG accumulation [126]. The thiazolidinedione pioglitazone, a PPAR agonist, has been shown to inhibit the mitochondrial pyruvate carrier in the liver [127], and also to attenuate hepatic pyruvate dehydrogenase activity in vitro [128], in turn, leading to reduced availability of carbohydrate-derived acetyl-CoA.

A reduction in glycolysis could thus be speculated to have beneficial effects on liver substrate metabolism. Reduced carbohydrate intake represents a physiologic way of reducing pyruvate availability within the liver (Figure 2). One human study interestingly showed by oral ^13^C-proprionate administration and NMR that 2 wk of a carbohydrate-reduced diet (5E% carbohydrate and 61E% fat) led to 31% lower hepatic TCA cycle flux in obese individuals, when compared with another group who ingested a control diet with 47E% carbohydrate and 36E% fat [129]. Despite the eucaloric design, a weight loss of 4.4 ± 1.4 kg was observed in the low carbohydrate group compared with 2.3 ± 1.7 kg in the other group, which is why different energy availability may also have contributed to the findings.

### Concluding remarks

In conclusion, the summarized evidence from eucaloric intervention studies suggests that lowering of carbohydrate intake may be equal to or more important than total calorie restriction in reducing liver TG content. Thus, even under conditions of energy balance, the cumulative findings suggest a clinically significant effect of reducing carbohydrate intake and increasing dietary fat (and/or protein intake) on hepatic TG content in obese individuals with steatosis, in particular, in individuals with insulin resistance. Based on human studies, the mechanisms by which reduced carbohydrate and increased fat intake leads to lower hepatic TG accumulation may relate to increased FA beta-oxidation and increased ketogenesis in the liver; metabolic adaptations mimicking the response to calorie restriction. Moreover, DNL in the liver is downregulated when dietary carbohydrate availability is decreased, also under eucaloric conditions. Less insulin availability to the liver by reduced carbohydrate and hence portal insulin appearance is also likely to regulate substrate metabolism in the liver toward increased FA utilization and less substrate deposition. The role of low carbohydrate dietary effects on insulin, glycogen, pyruvate, and potentially also acetyl-CoA in mediating the hepatic adaptations waiting to be assessed in human studies.

## Author contributions

The authors’ responsibilities were as follows—all authors: conceptualized and designed this review; AL, KNB-M, BK: contributed to the literature review and data interpretation; AL: drafted the manuscript; and all authors: reviewed and approved the final manuscript.

## Conflict of interest

AL reports financial support was provided by Novo Nordisk Foundation. KNB-M reports financial support was provided by Novo Nordisk Foundation. BK reports financial support was provided by Novo Nordisk Foundation. KNB-M reports financial support was provided by Independence Research Fund Denmark.

## Funding

AL was supported by a postdoctoral research grant from the Danish Diabetes Academy, funded by the Novo Nordisk Foundation, grant number NNF17SA0031406. KNB-M has received research funding from Novo Nordisk Foundation (Excellence grant NNF18OC0032330) and Independence Research Fund Denmark (0134-00176B). BK has received research funding from the Novo Nordisk Foundation (NNF 20OC0063744).
